# Lesion size as a prognostic factor in the antifungal treatment of pulmonary cryptococcosis: a retrospective study with chest CT pictorial review of 2-year follow up

**DOI:** 10.1186/s12879-023-08131-0

**Published:** 2023-03-14

**Authors:** Yu Yan, Yuxiao Wu, Qin Wang, Xiaodan Zhu, Huayin Li, Hongni Jiang

**Affiliations:** 1 Department of Pulmonary and Critical Care Medicine, Zhongshan Hospital, Fudan University, Shanghai, 200032 China; 2 Department of Pulmonary Medicine, Zhongshan Hospital (Xiamen), Fudan University, Xiamen, Fujian 361015 China

**Keywords:** Pulmonary cryptococcosis, Computed tomography, Pulmonary lesions, Prognostic factors, Cryptococcal capsular antigen

## Abstract

**Background:**

Pulmonary cryptococcosis (PC) is a fungal infection that can have a variable prognosis depending on several factors. The objective of this study was to analyse the characteristics of pulmonary lesions and identify prognostic factors in patients with PC who were human immunodeficiency virus (HIV) -negative and underwent antifungal treatment.

**Methods:**

The study enrolled patients diagnosed with PC who were negative for HIV. Symptoms, CT characteristics of pulmonary lesions, serum cryptococcal capsular antigen (CrAg) titre, underlying diseases, and duration of antifungal treatment were evaluated over a 2-year follow-up.

**Results:**

A total of 63 patients (40 men and 23 women) with a mean age of 50.4 years were included. Half of the patients (50.8%) were asymptomatic, and the most common symptoms were cough (44.4%), expectoration (27.0%), and fever (17.5%). Pulmonary lesions were mainly present in the peripheral and lower lobes of the lung, with 35 cases classified as nodular-type lesions and 28 cases classified as mass-type lesions. At the first, third, sixth, 12th, and 24th-month follow-ups, the median proportion of residual pulmonary lesions were 59.6%, 29.9%, 12.2%, 9.6%, and 0.0%, respectively. During antifungal treatment, the lesions of 33 patients achieved complete response, while the remaining 30 patients did not. Compared with the non-CR group, the CR group had a lower baseline serum CrAg titre (median, 1:20 vs 1:80, *P* < 0.01), smaller pulmonary lesion size (median area, 1.6 cm^2^ vs 6.3 cm^2^, *P* < 0.01), lower Hounsfield-units (HU) radiodensity (median, − 60.0 HU vs − 28.5 HU, *P* < 0.05), more nodular-type lesions (72.7% vs 36.7%, *P* < 0.01), and fewer air-bronchogram signs (18.2% vs 43.3%, *P* < 0.05). Multivariate logistic regression analysis showed that a larger lesion size on chest CT scans was associated with a lower likelihood of achieving complete response [OR: 0.89; 95% CI (0.81–0.97); *P* < 0.05].

**Conclusions:**

PC was more commonly observed in HIV-negative men, and chest CT scans mostly revealed nodular-type lesions. After antifungal treatment, patients with smaller lesions had a better prognosis.

## Background

Pulmonary cryptococcosis (PC) is a fungal infection with a global distribution that can affect both immunosuppressed and immunocompetent individuals [1–4]. It has a 20–70% mortality rate in patients infected with human immunodeficiency virus (HIV) [5]. In China, the mortality rate of patients with PC but negative for HIV is 0.88% [6]. The human pathogenic fungi primarily responsible for PC are *Cryptococcus neoformans* and *Cryptococcus gattii*, which are ubiquitous in the environment [2]. Inhalation of spores and desiccated yeast cells with a diameter of 1–5 μm by humans can lead to infection of the lower airways and pulmonary alveoli [7]. Alveolar macrophage phagocytosis triggers immune cells to participate in granuloma formation, resulting in subpleural nodules or lymphadenopathy in imaging studies [8]. Risk factors for PC include HIV infection, organ transplantation, malignancy, diabetes, liver cirrhosis, and long-term use of glucocorticoids or immunosuppressive agents [4]. PC can be asymptomatic in immunocompetent individuals, with some patients presenting with atypical symptoms such as cough, expectoration, and chest pain [9, 10]. Imaging forms of chest computed tomography (CT) can show nodules, masses, exudative shadows, tree-in-bud signs, cavities, and pleural effusion, which are often misdiagnosed as lung cancer or bacterial infections [11, 12].

Fluconazole is the first-line recommendation for the management of PC in recently published guidelines [13–15], and the treatment course is at least 3 months with slow regression of lesions. The evaluation of therapeutic efficacy is challenging due to the low positivity rate of microbial culture and a lack of specific biomarkers, and there is still a lack of corresponding follow-up studies. The majority of studies on treatment effectiveness are case reports, and there is a shortage of large sample studies. Some studies have a short follow-up period and define clinical improvement as treatment success without focusing on cure [16]. Therefore, this study aimed to conduct a retrospective analysis of the response of pulmonary lesions and related prognostic factors in patients with HIV-negative PC after antifungal therapy for up to 2 years.

## Methods and materials

### Patients

**Fig. 1:**
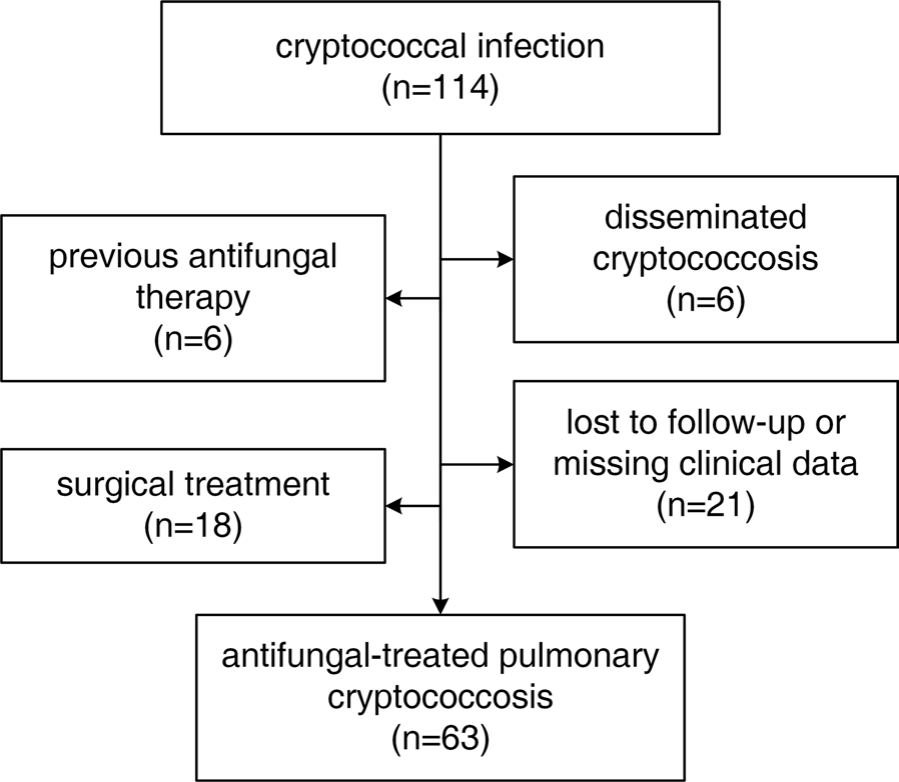
Patients enrolment

This retrospective study included patients with HIV-negative PC who were initially diagnosed and treated with antifungal drugs for at least 3 months at Zhongshan Hospital Affiliated to Fudan University from January 2018 to January 2021. Patients with incomplete clinical records, missing chest CT scans, disseminated cryptococcosis, and those who underwent surgical treatment were excluded (Fig. 1). A total of 63 patients were enrolled in this study. Data on demographic characteristics, clinical symptoms, comorbidities, medication records, baseline laboratory investigations such as complete blood count, liver and renal function tests, C-reactive protein (CRP), procalcitonin (PCT), erythrocyte sedimentation rate (ESR), cryptococcal capsular antigen (CrAg) titre, pathology results, and chest CT images were collected. Immunocompromised status was defined as the presence of severe diabetes mellitus with associated organ damage, liver cirrhosis, malignant disease (under immunosuppressive chemotherapy), or long-term use of steroids or immunosuppressive agents.

### Chest CT

**Fig. 2:**
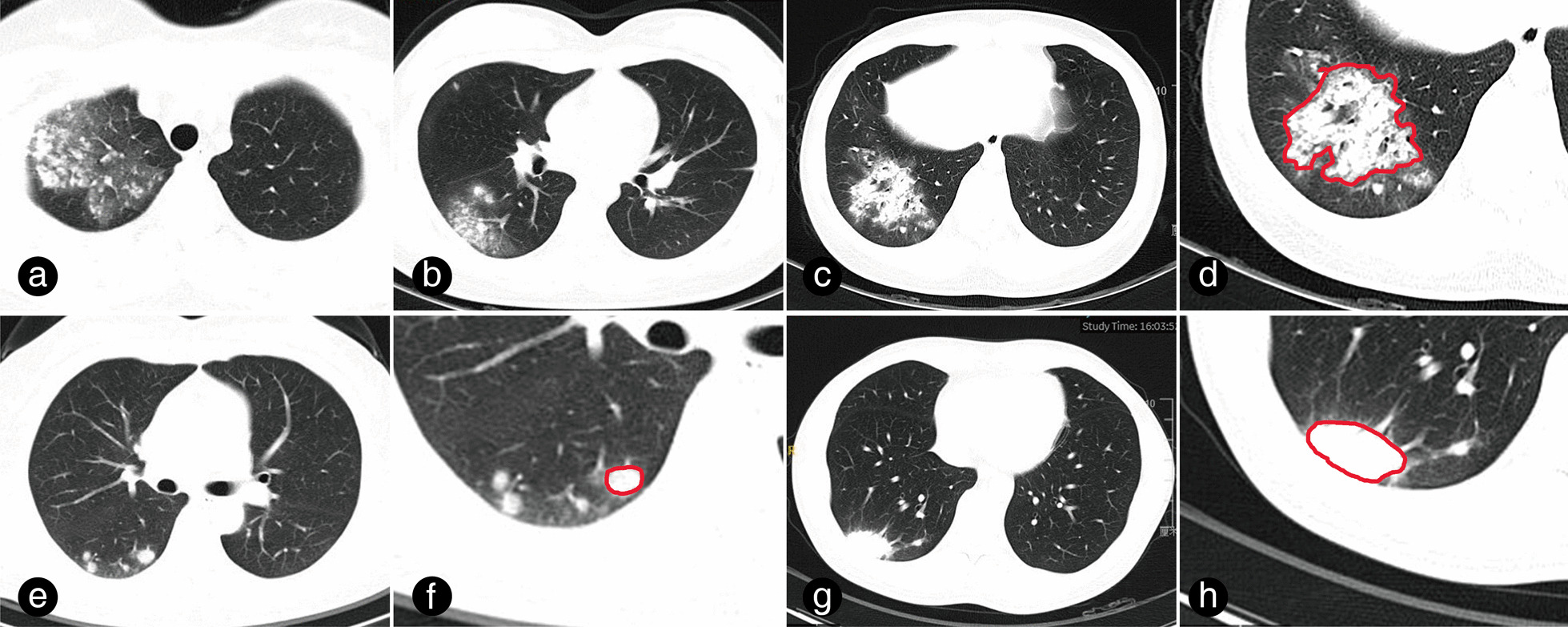
Measurement of the pulmonary lesion area and radiodensity. **a**–**d** The case of a 22-year-old female patient diagnosed with cryptococcal granuloma through endobronchial ultrasound-guided trans-bronchial needle aspiration (EBUS-TBNA) is presented. The patient exhibited multiple nodular and patchy lesions in the right lung **a**–**c**. The largest lesion was delineated with a red line in panel d, where its radiodensity (-173 HU) and area (14.12 cm^2^) were measured. **e**, **f** A 50-year-old female patient with multiple nodules in the right lower lobe and a serum CrAg titre of 1:5 was examined. The largest lesion was identified and its radiodensity (− 165 HU) and area (0.79 cm^2^) were measured using the same method as before. **g**, **h** A 44-year-old male patient with a serum CrAg titre of 1:2 and a mass lesion in the right lower lobe is described. The largest cross-sectional area of the lesion was marked by red line in panel h, where its radiodensity (− 13 HU) and area (5.06 cm^2^) were measured

The patients underwent a 64-slice CT scan of the chest while lying supine on the scanning table. The scans were performed after inhalation, during breath-holding, with a layer thickness of 1 to 5 mm. Two radiologists reviewed all chest CT scans retrospectively in a blinded manner, without access to any clinical information. The final interpretation was reached by consensus. Nodules were defined as lesions with a diameter ≤ 3.0 cm, while masses were defined as lesions with a diameter > 3.0 cm. The location, presence of cavitation, halo sign, air-bronchogram sign, and presence of single or multiple lesions were also documented. In addition, the area and radiodensity of the pulmonary lesion were measured using the Picture Archiving and Communication System (PACS) (Zhongshan Hospital, INFINITT SH CO., LTD), and radiodensity of lesions reported as the average CT number in Hounsfield Units (HU). For a single lesion, the largest cross-sectional area was measured, whereas, for multiple lesions, the largest cross-sectional area of the largest lesion was targeted (Fig. 2).

### Follow up

Clinical data was collected during follow-up visits of patients for up to 2 years following initial treatment, until May 2022. The recorded clinical data included treatment details such as drug type, dosage, and adverse reactions, serum CrAg titre, and the response of pulmonary lesions. The primary outcome was the regression of lesions, as determined by area and radiodensity at baseline, first, third, sixth, 12th, and 24th months. The secondary outcome was the synchronous transformation of serum CrAg titre.

### Outcome and group classification

**Fig. 3:**
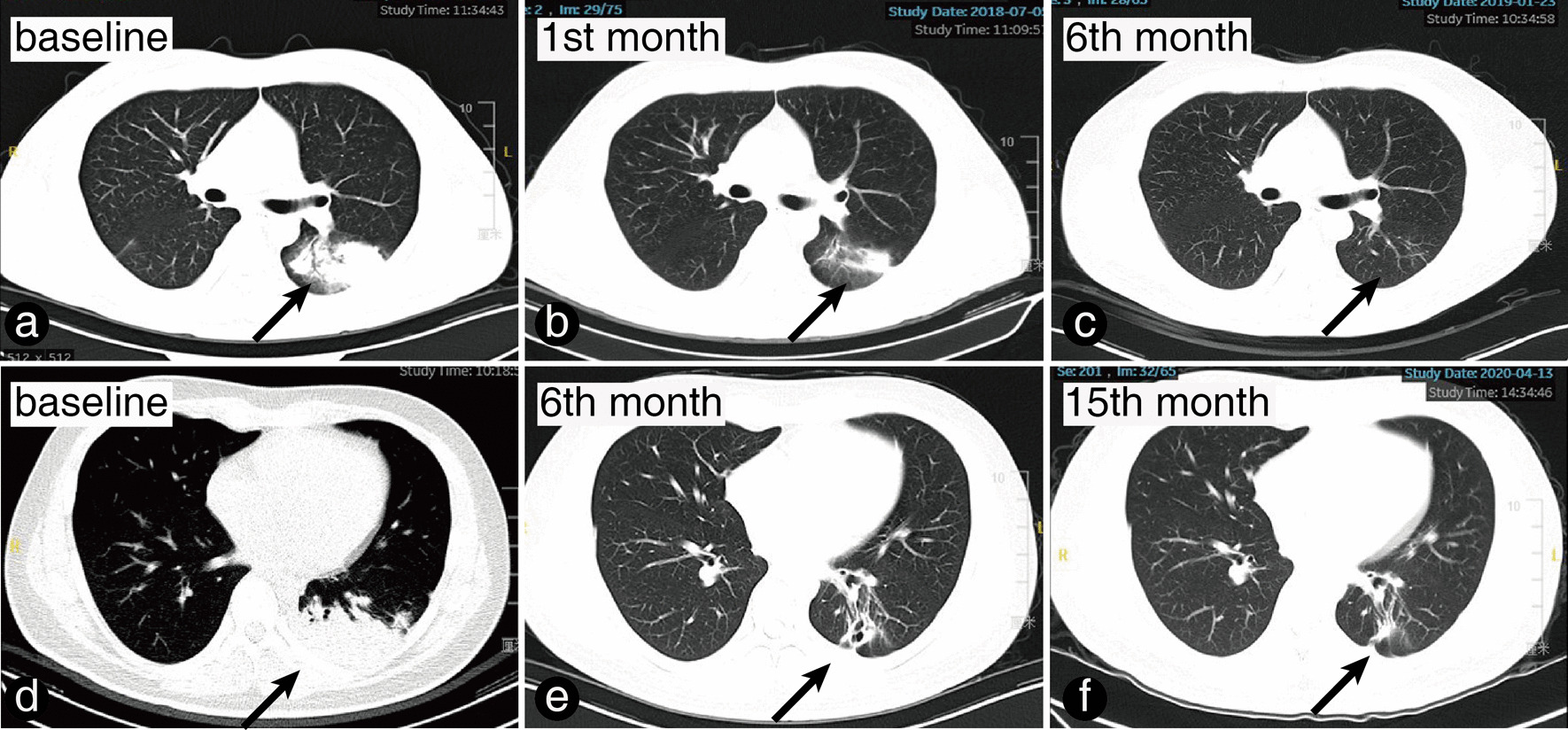
Follow-up lesion on chest CT and outcome group classification. **a**–**c** This case report is about a 55-year-old male patient who had a serum CrAg titre of 1:40 and a lesion in the left lung (**a**) (indicated by a black arrow). After treatment with fluconazole 400 mg/day, the lesion shrank (**b**) and ultimately disappeared (**c**), leading to the patient's assignment to the CR group. **d**–**f** In this case report, we present a 25-year-old male patient with pathologically confirmed PC and a mass-type lesion in the lower lobe of the left lung (**d**) (indicated by a black arrow) on chest CT. After treatment with fluconazole 400 mg/day, the lesion shrank (**e**) and eventually became a nodule (**f**). The patient was assigned to the non-CR group

Patients were classified into two groups based on the size of residual pulmonary lesions at their last visit. Those whose lesions disappeared or transformed into fibro stripes were assigned to the complete response (CR) group, while the remaining patients were assigned to the non-CR group. The latter group included patients with shrunken, stable, or progressive pulmonary lesions (Fig. 3).

### Statistical analysis

Continuous variables that followed a normal distribution are described as mean and standard deviation and were compared using Student’s *t* test. In order to conform to the normal distribution, CrAg titre was subjected to log transformation. Non-normally distributed continuous variables are presented as median (range) and were compared using *Mann–Whitney U* test. Categorical variables are reported as numbers and proportions and were compared between groups using either *chi-squared* test or *Fisher* exact test, as appropriate. Baseline variables that were deemed clinically relevant or showed a univariate relationship with the outcome (*P* < 0.1) were entered into a multiple logistic regression model. To ensure a parsimonious final model, only a limited number of variables were included given the number of events. A *P-*value < 0.05 (two-sided) was considered statistically significant. Statistical analyses were performed using SPSS v24.0 (IBM, Armonk, NY, USA).

## Results

### Demographics and clinical features

The demographic and clinical features of the patients are listed in Table 1. Among the 63 patients, 40 (63.5%) were male, and the mean age was 50.4 years. Sixteen patients were found to be immunocompromised, including those with severe diabetes, malignant disease, or prolonged use of glucocorticoids or immunosuppressive agents. Pathological biopsy was performed on 39 patients, of whom 29 tested positive for *Cryptococcus*. The majority of patients (90.5%) were serum CrAg-positive, with titres ranging from 1:2 to 1:1280. Thirty-two patients were asymptomatic, and among those who reported symptoms, cough, expectoration, and fever were the most common. Subpleural nodules were the most common type of pulmonary lesions observed, with most lesions involving the lower lobes. Single lesions were found in 38 patients, while 25 had multiple lesions. Nodular-type lesions were more frequently observed than mass-type lesions.

**Table 1:** The demographic and clinical features

	Number of Patients (n = 63)
Mean age, yr (range)	50.37 (17, 77)
Male: female	40: 23
Months of antifungal treatment, median (range)	6 (3, 20)
Immunocompromised, n (%)	16 (25.4)
Severe diabetes mellitus	1 (1.6)
Malignant diseases	7 (11.1)
Glucocorticoid or immunosuppressant therapy	11 (17.5)
Symptoms, n (%)	
Asymptomatic	32 (50.8)
Cough	28 (44.4)
Sputum	17 (27.0)
Fever	11 (17.5)
Chest pain	7 (11.1)
CrAg titre, median (range)	1:40 (0, 1:1280)
CT characteristics, n (%)	
CT number/HU, median (range)	− 45 (− 853, 55)
Area of lesions/cm^2^, median (range)	2.79 (0.09, 59.52)
Single lesion	38 (60.3)
Nodule-type	35 (55.6)
Mass-type	28 (44.4)
Halo sign	18 (28.6)
Air-bronchogram sign	19 (30.2)
Cavitation	13 (20.6)
Peripheral	49 (77.8)
Lower lobe	35 (55.6)

### Antifungal treatment

All patients received fluconazole as the initial treatment, with five patients receiving a daily dose of 200 mg, and 58 patients receiving a daily dose of 400–600 mg. Six patients changed their medicine during the treatment: four due to poor treatment response, one due to worsening renal insufficiency, and one due to diarrhoea. Five patients were switched to voriconazole, and one patient was switched to posaconazole. The median duration of antifungal therapy was 6 months (range: 3–20 months).

### Regression of lesions on chest CT scans

**Fig. 4:**
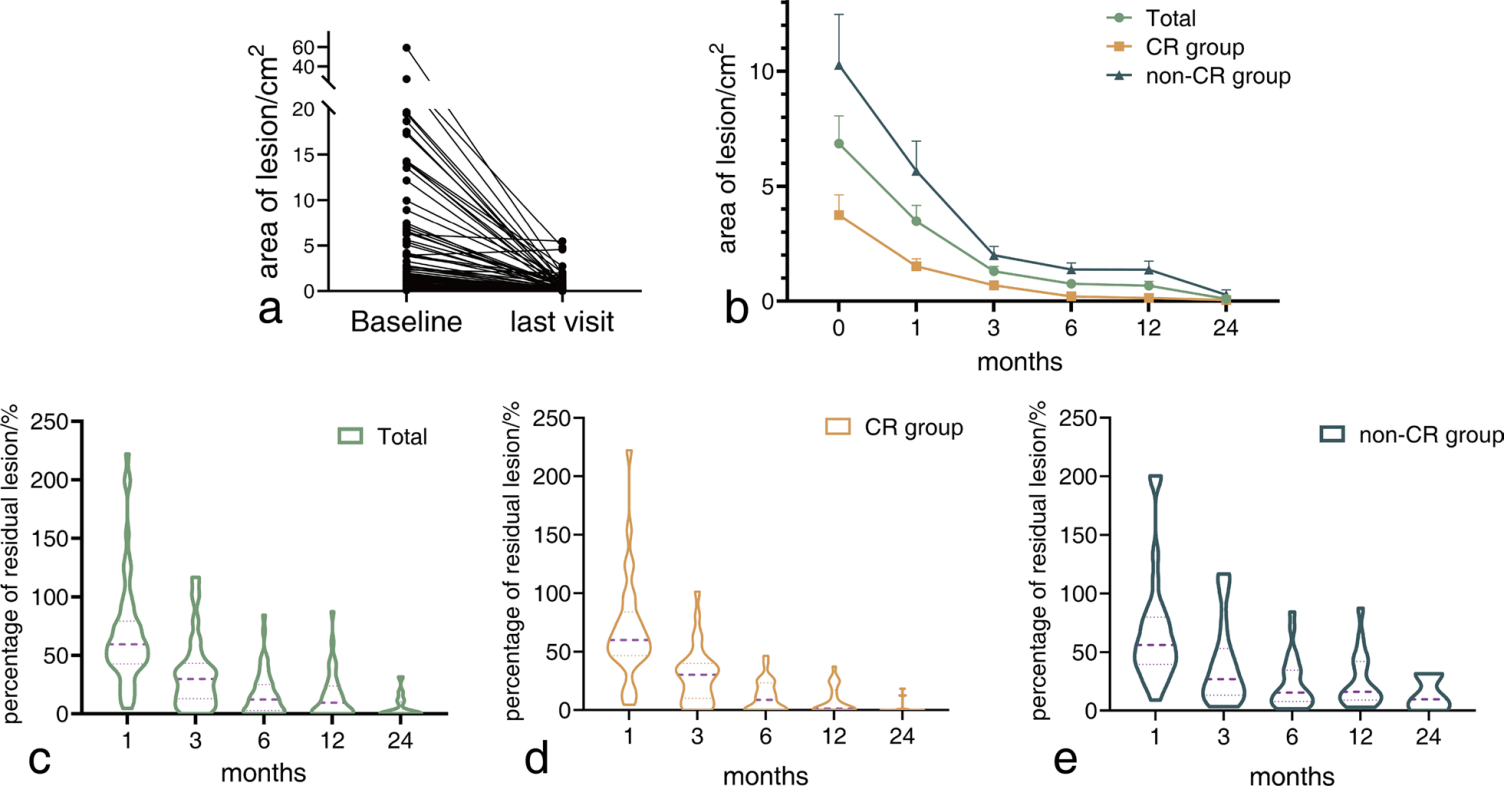
Follow-up of the area of pulmonary lesion. **a** Change in area of lesions at baseline and the last visit; **b** Change in area of residual lesions during follow-ups; **c** Percentage of the residual lesion in the total group; **d** Percentage of the residual lesion in the CR group (median: 1st month, 60.0%, n = 29; 3rd month, 30.4%, n = 31; 6th month, 8.9%, n = 27; 12th month, 1.3%, n = 22; 24th month, 0.0%, n = 11); **e** Percentage of the residual lesion in the non-CR group (median: 1st month, 56.3%, n = 26; 3rd month, 26.9%, n = 27; 6th month, 15.5%, n = 24; 12th month, 16.1%, n = 17; 24th month, 9.8%, n = 3)

During the follow-up period, the majority of patients exhibited a reduction in the size of their pulmonary lesions, with only one patient experiencing a slight increase in lesion size from 3.9 cm^2^ to 4.6 cm^2^ (Fig. 4a). The mean area of lesions also decreased over time (Fig. 4b) with a baseline of 6.9 ± 9.6 cm^2^ (n = 63), decreasing to 3.5 ± 5.1 cm^2^ (n = 55) at the 1st month, 1.3 ± 1.6 cm^2^ (n = 58) at the 3rd month, 0.8 ± 1.1 cm^2^ (n = 51) at the 6th month, 0.7 ± 1.2 cm^2^ (n = 39) at the 12th month, and 0.1 ± 0.2 cm^2^ (n = 14) at the 24th month. Additionally, the median percentage of residual lesion (Fig. 4c) also decreased, with values of 59.6%, 29.9%, 12.2%, 9.6%, and 0.0% at each respective follow-up time point. Based on the size of the residual pulmonary lesions at the last visit, 33 patients (52.4%) were classified in the CR group and 30 patients (47.6%) were classified in the non-CR group. In the CR group, lesions on chest CT scans nearly disappeared during the 6-12th-month period, while lesions were stable in the non-CR group during the same period (Fig. 4d, e). However, the data obtained from the 24th-month visit is unreliable due to the small number of patients who participated in this visit (11 in the CR group and 3 in the non-CR group).

### Dynamic change in radiodensity of lesions

**Fig. 5:**
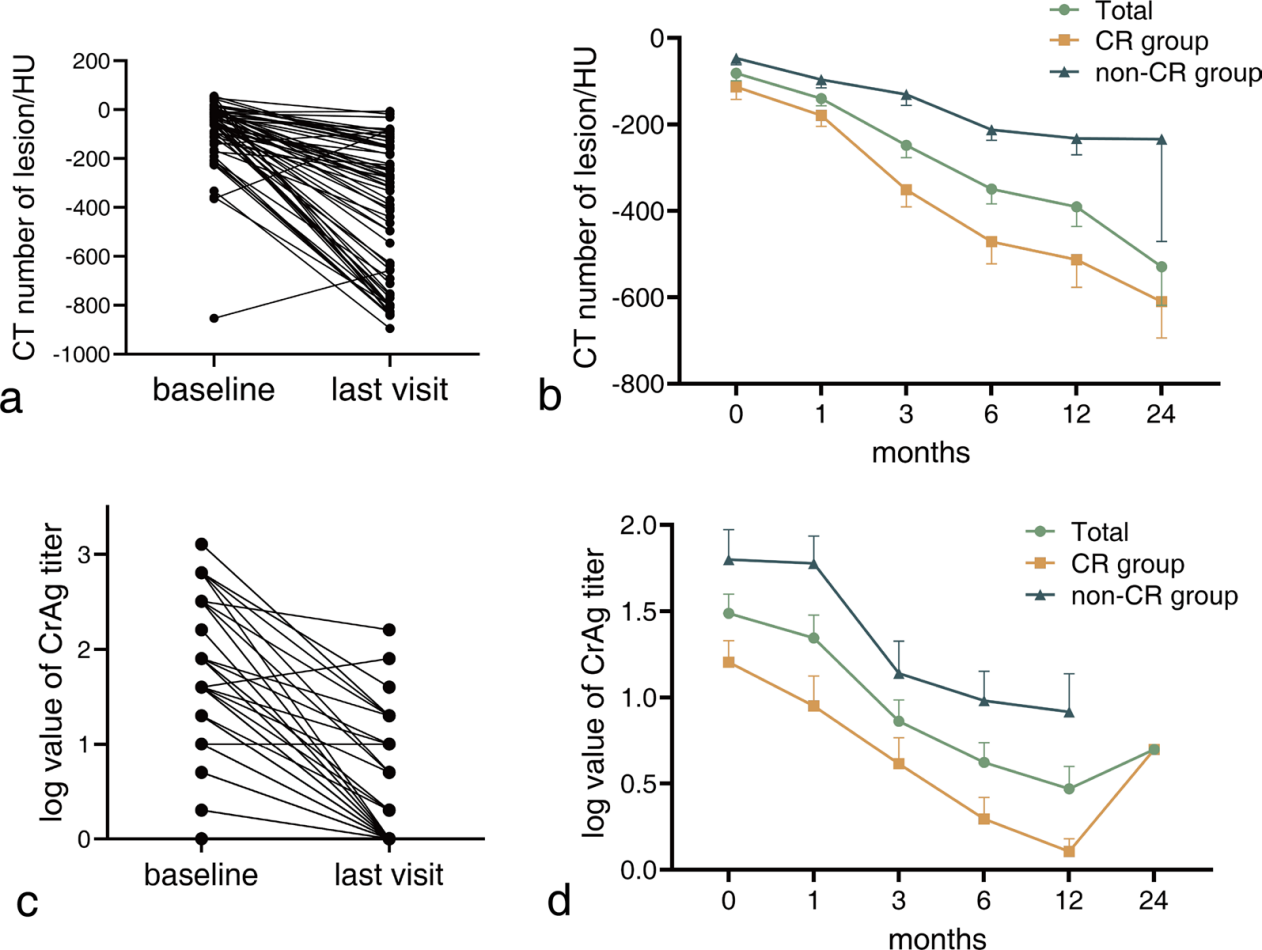
Follow-up of the radiodensity of pulmonary lesion and serum CrAg. **a** Change in radiodensity reported by CT number of lesions at baseline and the last visit; **b** Change in radiodensity reported by CT number of lesions during follow-ups; **c** Change in serum CrAg titre at baseline and the last visit; **d** Change in serum CrAg titre during follow-ups

The radiodensity of the lesions, reported by CT number on chest CT scans, decreased in the majority of patients (59/63), while it increased in four patients, three of whom had shrunken lesions and one had an enlarged lesion (Fig. 5a). Overall, the CT number tended to decrease (Fig. 5b) during the follow-up period, with a mean CT number of − 81.5 HU at baseline and a mean CT number of − 529.4 HU at the 24th month (n = 14). The mean CT numbers at the 1st, 3rd, 6th, and 12th months were − 139.8 HU (n = 55), − 248.2 HU (n = 58), − 349.4 HU (n = 51), and − 390.7 HU (n = 39), respectively.

### Dynamic change of serum CrAg titre

In this study, the baseline serum CrAg levels were evaluated in a cohort of patients. Of the 63 patients, 57 tested positive for baseline serum CrAg, while 6 were negative. The median CrAg titre was 1:40 (range, 0–1:1280). At the last visit, serum CrAg was tested for 55 patients, of whom 36 had negative results and 19 tested positive. Of the CrAg-negative patients, which included both baseline negative patients (n = 5) and positive-to-negative patients (n = 31). In contrast, among the remaining CrAg-positive patients, 18 had decreasing titres, with median titres decreasing from 1:40 to 1:20, accompanied by lesion reduction. However, one patient exhibited an increase in CrAg titre from 1:40 to 1:80, accompanied by lesion enlargement (Fig. 5c).

Notably, the serum CrAg titre tended to decrease during the follow-up period. Specifically, the log value of the CrAg titre declined from baseline (1.5 ± 0.9, n = 63) to the first month (1.3 ± 0.9, n = 42), the third month (0.9 ± 0.9, n = 49), the sixth month (0.6 ± 0.8, n = 44), and the twelfth month (0.5 ± 0.7, n = 29). Only one patient had their serum CrAg titre tested at the 24th month, and the log value was 0.7 (Fig. 5d).

### Univariate analysis of two outcome groups

The present study conducted a univariate analysis on two distinct outcome groups, with the results displayed in Table 2. The CR group exhibited lower baseline lesion area (median, 1.6 cm^2^ vs 6.3 cm^2^, *P* < 0.01), CT number (median, − 60 HU vs − 28.5 HU, *P* < 0.05), and CrAg titre (median, 1:20 vs 1:80, *P* < 0.01) when compared to the non-CR group. In addition, nodular-type lesions were more frequently observed in the CR group (*P* < 0.01), while air-bronchogram signs were less prevalent (*P* < 0.05) when compared to the non-CR group. However, cavitation lesions, halo signs, and single lesions were not statistically different between the two groups (all *P* > 0.05). Moreover, while the CR group exhibited lower levels of elevated neutrophils and ALT in the peripheral blood when compared to the non-CR group, these differences were not statistically significant.

**Table 2:** Univariate analysis of two outcome groups

	CR group	Non-CR group	*χ*^2^*/t*	*P*
Male: female	22:11	18:12	0.301	0.583
Age/yr (SD)	52.42 (13.27)	48.10 (16.13)	− 1.166	0.248
Immunocompromised, n (%)	8 (24.2)	8 (26.7)	0.049	0.825
Duration of treatment/months^a^	6 [3, 12]	6.5 [3, 20]		0.368
Baseline log value of CrAg (SD)	1.20 (0.71)	1.80 (0.95)	3.148	0.006
Baseline area of lesions^a^/cm^2^	1.59 [0.09, 19.47]	6.31 [0.41, 59.52]		0.001
Baseline CT number^a^/HU	− 60.0 [− 853, 54]	− 28.5 [− 364, 55]		0.037
Image type, n (%)				
Nodules	24 (72.7)	11 (36.7)	8.28	0.004
Masses	9 (27.3)	19 (63.3)		
Cavitation, n (%)	9 (27.3)	6 (20.0)	0.458	0.498
Halo sign, n (%)	10 (30.3)	8 (26.7)	0.102	0.750
Air-bronchogram sign, n (%)	6 (18.2)	13 (43.3)	4.72	0.030
Single lesion, n (%)	21 (63.6)	17 (56.7)	0.319	0.572
Pulmonary lobe, n (%)				
LU	7 (14.6)	2 (6.1)	3.659	0.454
LL	13 (27.1)	9 (27.3)		
RU	5 (10.4)	4 (12.1)		
RM	5 (10.4)	1 (3.0)		
RL	18 (37.5)	17 (51.5)		
Baseline laboratory tests, n(%)				
Lowered RBC	9 (29.0)	12 (40.0)	0.812	0.367
Elevated WBC^b^	2 (6.3)	6 (20.0)		0.141
Elevated N	3 (9.4)	8 (26.7)	3.172	0.075
Lowered L^b^	5 (16.1)	4 (13.3)		1.000
Elevated PLT	6 (19.4)	8 (26.7)	0.461	0.497
Elevated ESR	3 (30.0)	7 (35.0)	0.075	0.784
Elevated CRP	14 (50.0)	14 (46.7)	0.064	0.800
Elevated ALT^b^	1 (3.1)	5 (16.7)		0.099
Elevated AST^b^	1 (3.1)	1 (3.3)		1.000
Lowered Alb^b^	1 (3.1)	3 (10.0)		0.346
Lowered Glob^b^	1 (3.1)	1 (3.3)		1.000
Elevated LDH	4 (12.9)	6 (20.7)	0.654	0.419

*LU* left upper lobe; *LL* left lower lobe; *RU* right upper lobe; *RM* right middle lobe; *RL* right lower lobe; *RBC* red blood cells; *WBC* white blood cells; *N* neutrophil; *L* lymphocyte; *PLT* platelet; *ESR* erythrocyte sedimentation rate; CRP, C-reactive protein; ALT, alanine aminotransferase; AST, aspartate aminotransferase; Alb, albumin; Glob, globulin; LDH, lactate dehydrogenase

^a^Described as median [range] and compared with *Mann–Whitney U* test

^b^Compared with *Fisher* exact test

### Multivariate logistic regression model

In the present study, variables with a *P* value less than 0.1 in the univariate analysis were screened, along with clinical significance and detection efficiency, to determine the factors to include in the multivariate logistic regression analysis. Four variables namely, baseline lesion area, baseline CT number, air-bronchogram sign, and elevated N, were included in the multivariate logistic regression analysis (Table 3). The method was chosen to perform subsequent analysis, and ultimately, the baseline area of lesions was included in the regression equation. It was observed that the larger the size of the lesion, the more difficult it was to achieve CR in terms of chest CT scans [odds ratio (OR): 0.89; 95% confidence interval (CI) 0.81–0.97; *P* < 0.05].

**Table 3:** Multivariate logistic regression model

	OR (95% CI)	*P*
Baseline area of lesions	0.89 (0.81–0.97)	0.013
Baseline CT number	…	0.330
Elevated N	…	0.248
Air-bronchogram sign	…	0.903

*OR* Odds ratio; *95% CI* 95% confidence interval

## Discussion

This retrospective study aimed to investigate the characteristics of pulmonary lesions in patients with PC who were not infected with HIV and to describe the dynamic changes after antifungal therapy. The majority of the pulmonary lesions appeared in the lower lobes of the lung, with peripheral single nodules being the most common, consistent with inhalation-type infection lesions. Previous studies have shown differences in lesion characteristics between immunocompetent and immunocompromised patients. In a study by Wang et al., nodular lesions (97.6%) and single lesions (59.5%) were more common in immunocompetent patients, while consolidation (57.7%), multiple lesions (92.3%), air-bronchogram signs (73.1%), and cavities (34.6%) were the main characteristics in patients who were immunocompromised [17]. Chang et al. showed that an evolution to cavitary lesions often occurs in patients who are immunocompromised, which represents a more aggressive disease nature [18]. Our study, however, found no difference in cavitary lesions between the two outcome groups, and the air-bronchogram signs were more common in the poor outcome group (*P* < 0.05). Half of the patients were asymptomatic, and the most common symptoms were cough, expectoration, and fever, which were consistent with the results of previous studies [9, 10].

Invasive fungal disease (IFD) is categorized by the European Organization for Research and Treatment of Cancer/Invasive Fungal Infections Cooperative Group and the National Institute of Allergy and Infectious Diseases Mycoses Study Group (EORTC/MSG) as ‘proven’, ‘probable’, and ‘possible’ [19]. Proven IFD requires microscopic analysis of sterile material, cultures from blood/sterile samples, or positive CrAg in cerebrospinal fluid or blood. The category of proven IFD can apply to any patient, regardless of whether the patient is immunocompromised [20]. In clinical practice, CrAg has significant value as a specific biomarker in the diagnosis of cryptococcosis and evaluation of therapeutic efficacy [21]. One study showed that the sensitivity, specificity, positive predictive value, and negative predictive value of CrAg in lung tissue and serum samples were 100%, 97%, 89%, 100%, and 37.5%, 100%, 100%, and 76%, respectively [22]. Our study showed that the serum CrAg titre changed synchronously with the size of the lesion. During treatment, CrAg increased from 1:40 to 1:80, and the lesion area increased from 3.90 cm^2^ to 4.56 cm^2^ in one patient, while both CrAg and lesion area decreased for 49 patients. Several studies have also shown that CrAg decreases with effective treatment, which can reflect the process of reducing lesions [18, 23], consistent with the results of our study.

Meanwhile, false negative and false positive results of CrAg testing are worth attention. In our study, six patients were found to be CrAg-negative but confirmed by pathology. In addition, it has been reported that CrAg cross-reacts with other fungi and immunoglobulins (especially rheumatoid factor), resulting in false-positive results [24]. These findings highlight the potential limitations of relying solely on CrAg testing for the diagnosis of cryptococcosis and emphasize the importance of incorporating clinical and pathological information in the diagnostic algorithm. Further studies are warranted to elucidate the factors contributing to the discordant results and to improve the accuracy of CrAg testing in the diagnosis of cryptococcosis.

Our study found that a larger initial lesion area was associated with a worse prognosis, possibly due to the fungal quantity, virulence, and host inflammatory response. Among the 29 cases confirmed by pathology, 15 exhibited nodular-type lesions, and 14 demonstrated mass-type lesions. The most common pathological manifestation for both lesion types was the presence of granulomatous lesions (6 in nodular-type lesions and 8 in mass-type lesions), followed by inflammatory cell infiltration with fibrous tissue hyperplasia (5 in nodular-type lesions and 5 in mass-type lesions), and absence of inflammatory cell infiltration (4 in nodular-type lesions and 1 in mass-type lesions). Cases with large lesions showed a strong correlation with the inflammatory response in the body, and the pathological manifestations were mostly inflammatory cell infiltration, with or without granuloma formation.

This study is subject to certain limitations that may affect the generalizability of its findings. First, the retrospective design of the study is inherently prone to bias, particularly due to the lack of complete follow-up data, especially at the 24th-month follow-up where only 14 patients underwent chest CT imaging and one patient had CrAg titre testing. Secondly, the sample size was relatively small, with only 63 patients included in the study. Lastly, all cases were obtained from a single center, which raises the possibility of center-specific bias. Hence, further studies with larger sample sizes and multi-center designs are warranted to validate the conclusions of this study.

## Conclusions

The current study analysed the CT characteristics of pulmonary lesions in HIV-negative patients with PC and identified lesion size as a prognostic factor. The majority of pulmonary lesions observed were of the nodular type and predominantly located in the lower and peripheral regions of the lung. It was found that achieving CR of pulmonary lesions, as indicated by chest CT scans, was more challenging in cases where lesions were larger in size. This highlights the importance of closely monitoring the response of larger-sized lesions and administering timely and appropriate antifungal therapy. Overall, the findings of this study suggest that clinicians should pay careful attention to the size of pulmonary lesions, as it can affect treatment outcomes in patients with pulmonary cryptococcosis.

## Data Availability

All data generated or analysed during this study are included in this published article.

## Abbreviations

HIV: Human immunodeficiency virus
PC: Pulmonary cryptococcosis
CT: Computer tomography
HU: Hounsfield unit
CrAg: Cryptococcal capsular antigen
CRP: C-reactive protein
PCT: Procalcitonin
ESR: Erythrocyte sedimentation rate
CR: Complete response
N: Neutrophil
ALT: Alanine aminotransferase
IFD: Invasive fungal disease
OR: Odds ratio
95% CI: 95% Confidence interval

## References

[1] Molloy SF, Kanyama C, Heyderman RS, Loyse A, Kouanfack C, Chanda D, Mfinanga S, Temfack E, Lakhi S, Lesikari S (2018). Antifungal combinations for treatment of Cryptococcal meningitis in Africa. N Engl J Med.

[2] Zavala S, Baddley JW (2020). Cryptococcosis. Semin Respir Crit Care Med.

[3] Chang CC, Sorrell TC, Chen SC (2015). Pulmonary Cryptococcosis. Semin Respir Crit Care Med.

[4] Setianingrum F, Rautemaa-Richardson R, Denning DW (2019). Pulmonary cryptococcosis: a review of pathobiology and clinical aspects. Med Mycol.

[5] Brown GD, Denning DW, Gow NA, Levitz SM, Netea MG, White TC (2012). Hidden killers: human fungal infections. Sci Transl Med..

[6] Chen LA, She DY, Liang ZX, Liang LL, Chen RC, Ye F, Li YP, Zhou Y, Chen XH, Fang SF (2021). A prospective multi-center clinical investigation of HIV-negative pulmonary cryptococcosis in China. Zhonghua Jie He He Hu Xi Za Zhi.

[7] Ballou ER, Johnston SA (2017). The cause and effect of Cryptococcus interactions with the host. Curr Opin Microbiol.

[8] Maziarz EK, Perfect JR (2016). Cryptococcosis. Infect Dis Clin North Am.

[9] Hou X, Kou L, Han X, Zhu R, Song L, Liu T (2019). Pulmonary cryptococcosis characteristics in immunocompetent patients: a 20-year clinical retrospective analysis in China. Mycoses.

[10] Yamakawa H, Yoshida M, Yabe M, Baba E, Okuda K, Fujimoto S, Katagi H, Ishikawa T, Takagi M, Kuwano K (2015). Correlation between clinical characteristics and chest computed tomography findings of pulmonary cryptococcosis. Pulm Med.

[11] Guimarães MD, Marchiori E, Meirelles GS, Hochhegger B, Santana PR, Gross JL, Bitencourt AG, Boonsirikamchai P, Godoy MC (2013). Fungal infection mimicking pulmonary malignancy: clinical and radiological characteristics. Lung.

[12] Qu J, Zhang X, Lu Y, Liu X, Lv X (2020). Clinical analysis in immunocompetent and immunocompromised patients with pulmonary cryptococcosis in Western China. Sci Rep.

[13] Izumikawa K, Kakeya H, Sakai F, Shibuya K, Sugita T, Takazono T, Takata T, Tashiro M, Teruya K, Nakamura S (2020). Executive summary of JSMM clinical practice guidelines for diagnosis and treatment of cryptococcosis 2019. Med Mycol J.

[14] Chang CC, Hall V, Cooper C, Grigoriadis G, Beardsley J, Sorrell TC, Heath CH (2021). Consensus guidelines for the diagnosis and management of cryptococcosis and rare yeast infections in the haematology/oncology setting, 2021. Intern Med J.

[15] Baddley JW, Forrest GN (2019). Cryptococcosis in solid organ transplantation-guidelines from the american society of transplantation infectious diseases community of practice. Clin Transplant.

[16] Suwatanapongched T, Sangsatra W, Boonsarngsuk V, Watcharananan SP, Incharoen P (2013). Clinical and radiologic manifestations of pulmonary cryptococcosis in immunocompetent patients and their outcomes after treatment. Diagn Interv Radiol.

[17] Wang DX, Zhang Q, Wen QT, Ding GX, Wang YG, Du FX, Zhang TY, Zheng XY, Cong HY, Du YL (2022). Comparison of CT findings and histopathological characteristics of pulmonary cryptococcosis in immunocompetent and immunocompromised patients. Sci Rep.

[18] Chang WC, Tzao C, Hsu HH, Lee SC, Huang KL, Tung HJ, Chen CY (2006). Pulmonary cryptococcosis: comparison of clinical and radiographic characteristics in immunocompetent and immunocompromised patients. Chest.

[19] De Pauw B, Walsh TJ, Donnelly JP, Stevens DA, Edwards JE, Calandra T, Pappas PG, Maertens J, Lortholary O, Kauffman CA (2008). Revised definitions of invasive fungal disease from the European organization for research and treatment of cancer/invasive fungal infections cooperative group and the national institute of allergy and infectious diseases mycoses study group (EORTC/MSG) consensus group. Clin Infect Dis.

[20] Donnelly JP, Chen SC, Kauffman CA, Steinbach WJ, Baddley JW, Verweij PE, Clancy CJ, Wingard JR, Lockhart SR, Groll AH (2020). Revision and update of the consensus definitions of invasive fungal disease from the European organization for research and treatment of cancer and the mycoses study group education and research consortium. Clin Infect Dis.

[21] Cheng KB, Wu ZH, Liang S, Li HP, Xu JF (2021). Associations of serum cryptococcal antigen with different of clinical characteristics: a comprehensive analysis of 378 pulmonary cryptococcosis patients. Ann Palliat Med.

[22] Liaw YS, Yang PC, Yu CJ, Chang DB, Wang HJ, Lee LN, Kuo SH, Luh KT (1995). Direct determination of cryptococcal antigen in transthoracic needle aspirate for diagnosis of pulmonary cryptococcosis. J Clin Microbiol.

[23] Fisher JF, Valencia-Rey PA, Davis WB (2016). Pulmonary cryptococcosis in the immunocompetent patient-many questions, some answers. Open Forum Infect Dis.

[24] Cheng MP, Nguyen TT, Parkes LO, Dufresne PJ, Sheppard DC (2017). Cross-reacting *Ustilago*
*maydis* causing false-positive cryptococcal antigen test results. J Clin Microbiol.

